# Variation in Direct Access to Tests to Investigate Cancer: A Survey of English General Practitioners

**DOI:** 10.1371/journal.pone.0159725

**Published:** 2016-07-22

**Authors:** Brian D. Nicholson, Jason L. Oke, Peter W. Rose, David Mant

**Affiliations:** Nuffield Department of Primary Care Health Sciences, University of Oxford, Oxford, Oxfordshire, OX26GG, United Kingdom; University Medical Center of Princeton/Rutgers Robert Wood Johnson Medical School, UNITED STATES

## Abstract

**Background:**

The 2015 NICE guidelines for suspected cancer recommend that English General Practitioners have direct access to diagnostic tests to investigate symptoms of cancer that do not meet the criteria for urgent referral. We aimed to identify the proportion of GPs in England with direct access to these tests.

**Methods:**

We recruited 533 English GPs through a national clinical research network to complete an online survey about direct access to laboratory, radiology, and endoscopy tests in the three months leading up to the release of the 2015 NICE guidance. If they had direct access to a diagnostic test, GPs were asked about the time necessary to arrange a test and receive a report. Results are reported by NHS sub-region and, adjusting for sampling, for England as a whole.

**Results:**

Almost all GPs reported direct access to x-ray and laboratory investigations except faecal occult blood testing (54%, 95% CI 49–59%) and urine protein electrophoresis (89%, 95% CI 84–92%). Fewer GPs had direct access to CT scans (54%, 95% CI 49–59%) or endoscopy (colonoscopy 32%, 95% CI 28–37%; gastroscopy 72%, 95% CI 67–77%). There was significant variation in direct access between NHS regions for the majority of imaging tests—for example, from 20 to 85% to MRI. Apart from x-ray, very few GPs (1–22%) could access radiology and endoscopy within the timescales recommended by NICE. The modal request to test time was 2–4 weeks for routine radiology and 4–6 weeks for routine endoscopy with results taking another 1–2 weeks.

**Conclusion:**

At the time that the 2015 NICE guideline was released, local investment was required to not only provide direct access but also reduce the interval between request and test and speed up reporting. Further research using our data as a benchmark is now required to identify whether local improvements in direct access have been achieved in response to the NICE targets. If alternative approaches to test access are to be proposed they must be piloted comprehensively and underpinned by robust effectiveness data.

## Background

Early definitive investigation for cancer in primary care is associated with improved survival [1]. Patients express a preference for early investigation even when cancer risk is as low as 1% [2]. However, while early investigation in primary care can lead to more cancers being detected at a stage amenable to curative treatment [3, 4], it can also lead to delay in specialist referral, even in cases where symptoms meet urgent referral criteria [5]. The publication of “Achieving World-Class Cancer Outcomes: as Strategy for England 2015–2020” by the Independent Cancer Taskforce has highlighted international differences in recommended approaches to primary care investigation of cancer-related symptoms and the need for underpinning evidence [6].

The 2015 NICE guidance for suspected cancer is based on evidence from UK primary care. It recommends that GPs investigate or refer any patient who has a 3% risk of cancer (and sometimes a lower risk—see S1 Appendix) [7]. It points out that GPs must recognise and investigate “low-risk-but-not-no-risk” symptoms of cancer such as fatigue and weight loss to increase the number of cases detected through primary care, as patients referred from primary care with traditional high risk (of over 5%) or “red-flag” symptoms such as haemoptysis and rectal bleeding account for the minority of cancers detected [7]. In order to facilitate this change in clinical practice, NICE also recommended that GPs are given direct and rapid access to diagnostic tests including laboratory tests (Ca125, testing for occult blood in faeces, Full Blood Count), radiology (X-Ray, CT, MRI) and ultrasound, and endoscopy of the gastro-intestinal tract for patients who do not meet the criteria for an urgent (within 2 weeks) referral to a specialist but who do have symptoms warranting further urgent investigation (see S1 Appendix). Examples include Gastroscopy within 2 weeks for patients aged 55 and over with weight loss and upper abdominal pain, and ultrasound (USS) within 2 weeks for adults with an unexplained lump that is increasing in size (NICE 2015b).

Direct access to imaging and endoscopy has been shown to improve care in a number of countries. For example, a Dutch study has demonstrated that direct access colonoscopy reduces diagnostic delay and specialist workload without markedly increasing the volume of procedures done [8]. A Danish study has shown that direct access to CT Thorax did not increase test use but significantly reduced specialist time per patient and with high staff acceptability [9]. And an English study has demonstrated that GP practices had lower emergency admission and mortality for oesophago-gastric cancer if they had higher rates (implying better access to) gastroscopy [10].

This study aimed to assess the extent to which these NICE guidelines on the investigation of cancer symptoms can be implemented in England by surveying the extent to which GPs have direct access to the specified tests. The survey was done in the months leading up to the release of the 2015 NICE guidance.

## Methods

### Survey Development

The survey was adapted from the International Cancer Benchmarking Partnership Module 3 (ICBP3) questionnaire [11] by the lead author using Survey Monkey (www.surveymonkey.com). To ensure face validity, it was piloted by 17 GPs (including 6 academic GPs) from England and refined in response to the comments received (data not shown). Throughout the survey “direct access” was defined as “a test that a GP can request without having to speak to a specialist first” and was distinct from 2 week wait referral, one-stop clinics, or specialist referral [7].

The final survey included questions about: 1) practice characteristics (name, postcode, population, CCG); 2) access to NHS test providers (e.g. hospital trusts), NHS test centres (e.g. hospitals), and private test providers; 3) access to laboratory tests (Ca125, Calcium, Erythrocyte Sedimentation Rate, Faecal Occult Blood Testing, Full Blood Count, Liver Function Tests, Plasma Viscosity, Prostate Specific Antigen, Renal Function, Serum Protein Electrophoresis, Urine Protein Electrophoresis); 4) access to radiology (X-Ray, CT, MRI, Ultrasound, PET scanning, Mammography); 5) access to endoscopy (Gastroscopy, Flexible Sigmoidoscopy, Colonoscopy); 6) communication of results; 7) clinical guidance, and; 8) access to specialist advice regarding the investigation or referral of suspected cancer (S1 Survey Instrument).

Before recruitment commenced, the survey gained approval from the University of Oxford’s Central University Research Ethics Committee (MS-IDREC-C1-2015-066).

### Survey Participants

Recruitment began on April 13^th^ 2015 and remained open until 17^th^ July 2015. Test access is not dependent on individual GP characteristics but on the diagnostic services commissioned by local Clinical Commissioning Groups (CCGs). We therefore sought to recruit a geographically diverse sample of GPs through the NIHR Clinical Research Network (CRN) for Primary Care which has participant GP practices (with a commitment to provide reliable survey data) across all English regions [12]. As GPs in boundary areas may have access to services commissioned by more than one local CCG, we could not simply approach one practice in each Clinical Commissioning Group area. Instead, the NIHR CRN Coordinating Centre asked local regional leads to identify purposively and approach a sample of GPs that were geographically representative and likely to be willing to complete a demanding and complex survey about local NHS provision with care and precision. The lead researcher responded to queries from CRN leads and GPs by email. GPs were invited to give consent on the first page of the survey, and were not reimbursed for their time.

### Analysis

For each laboratory and radiology/imaging test we calculated: 1) the regional variation in the level of access; 2) the time to taken for the tests to be performed and reported; 3) the degree to which the NICE recommendations on direct access could be met. Survey responses were presented as absolute numbers and proportions of GPs reporting direct access to laboratory, radiology, and endoscopy and were aggregated for NHS England as a whole and stratified into the 13 NHS England sub-regions formed in April 2015. NHS England level estimates of the proportions of GP’s with test access were weighted by the sub-region sampling fraction (number of practices in sub-region/number of practices sampled) to adjust for the under- and over-sampling in some sub-regions. Fisher’s exact test was used to test for differences in access across sub-regions and two-sided *p*-values were calculated using Monte-Carlo simulation (10,000 replications). A *p-*value of <0.05 was deemed significant.

We applied two internal validity checks: 1) we asked for the same information in different parts of the survey and checked for consistency of response; 2) we asked about direct access to two tests (mammography and PET scans) for which there is no direct access in the UK NHS. All data from GPs who failed these internal validity checks were excluded from the analysis. Data from GPs who gave incomplete responses to questions were also excluded.

In the survey, GP’s were asked to report the time it took for routine and urgent laboratory or imaging test requests to be implemented (the request to test or R-T interval) and the time between the test being performed and the result being reported back to the GP (the test to report or T-R interval). Rather than give an exact time, respondents were asked to choose one of 7 options to describe the time interval, as either: 1) same day; 2) less than 1 week; 3) between 1 and 2 weeks; 4) 2 to 4 weeks; 5) 4 to 6 weeks; 6) longer than 6 weeks, and; 7) Didn’t have access or didn’t know. Request to test and test to report intervals were combined to form “request to report” intervals (R-R) for each test. Access timing data was dichotomised as either less than or equal to 2 weeks, or more than 2 weeks. Two weeks was chosen for the laboratory tests as this is the longest interval recommended for direct test access by NICE (S1 Appendix) [7]. This analysis was conducted to further explore the association between the R-T and T-R interval. For the R-R interval to qualify as less than 2 weeks a respondent had to report that their request to test and test to report intervals were either both “same day”, one “same day” and the other “less than 1 week”, both “less than 1 week” or one “1–2 weeks” and the other “same day”. All other combinations were categorised as “longer than 2 weeks”. The association between the categorical time variables of R-T and T-R were investigated by cross-tabulating responses in 5x5 matrices or grids (S2 Appendix).

In addition, we examined the proportions of GP’s who reported being able to meet current NICE guidelines for timely access to tests via routine or urgent direct access pathways for the investigation of cancer. In practice, GPs use routine and urgent referrals guided by their clinical suspicion of serious disease, but there is no national standardised definition of the duration of a routine or urgent test in clinical practice. In line with NICE, we calculated the proportion of GP’s who met the 48 hour recommendation for direct access to X-ray or ultrasound and the 2 week threshold for direct access to X-ray, Ultrasound, CT, MRI and Gastroscopy to include either routine or urgent referrals. A R-R interval of less than or equal to 48 hours was defined as both R-T and T-R being “same day”, and access was categorised into “< = 2 week” or “> 2 weeks” as before. We also report the proportion of GPs reporting different methods of test communication. All analyses were performed in Microsoft Excel, Stata (version 12), and R version 3.2.2 (R Core Team, 2015) and the R package survey [13].

## Results

### Number of GP respondents

The NIHR network had active contact with 1282 GPs during the study (16% of all GPs registered with the NIHR Research Network); 947 GPs (74%) opened the electronic survey and 533 (42%) completed it. Of those completing the survey, 389 (73%) gave full consent for making the data public, 137 (26%) consented but opted out of sharing practice level identifiable data, and seven (1%) chose not to give consent. Of the 526 GPs giving consent, 511 completed each part of every question relating to each test they reported having direct access to, and so were included in the final analysis.

### Number of GPs per region

The sub-regions of “Cheshire and Greater Manchester” and “Lancashire and Greater Manchester” were combined into “Cheshire, Lancashire, and Greater Manchester” because of their geographical proximity and low response rates (3 and 11 respectively). The number of GP respondents in the 12 remaining regions ranged from 14 to 68 (median 41, IQR 36.25–47.75) (Table 1).

**Table 1:** Proportion of English GPs with Direct Access to Laboratory and Imaging Tests for Cancer Investigation by Region.

TEST	ENGLAND	REGION(%) n (%)												*p*-value
	N = 511													
	n (%, 95% C.I)	C.Mid	East	Lndn	Manc	N.East	N.Mid	Sth.C	Sth.E	Sth.W	Wessx	W.Mid	YksH	
		(N = 34)	(N = 39)	(N = 68)	(N = 14)	(N = 40)	(N = 47)	(N = 63)	(N = 50)	(N = 47)	(N = 30)	(N = 42)	(N = 37)	
***Laboratory Testing***														
**Ca-125**	507 (99, 98–100)	34 (100)	39 (100)	67 (99)	14 (100)	40 (100)	47 (100)	61 (97)	50 (100)	47 (100)	30 (100)	42 (100)	36 (97)	0.78
**Erythrocyte Sedimentation Rate or Plasma Viscosity**	506 (99, 98–100)	34 (100)	39 (100)	68 (100)	14 (100)	40 (100)	47 (100)	63 (100)	50 (100)	44 (94)	30 (100)	42 (100)	35 (95)	0.01
**Faecal Occult Blood Test**	270 (54, 49–59)	21 (62)	22 (56)	47 (69)	7 (50)	17 (42)	22 (47)	31 (49)	17 (34)	21 (45)	24 (80)	24 (57)	17 (46)	<0.01
**Prostate Specific Antigen**	510 (100, 98–100)	34 (100)	39 (100)	68 (100)	14 (100)	40 (100)	47 (100)	63 (100)	50 (100)	47 (100)	30 (100)	42 (100)	36 (97)	0.23
**Serum Protein Electrophoresis**	502 (97, 94–99)	33 (97)	38 (97)	66 (97)	13 (93)	39 (98)	47 (100)	63 (100)	50 (100)	47 (100)	30 (100)	41 (98)	35 (95)	0.25
**Urine Protein Electrophoresis**	465 (89, 84–92)	30 (88)	34 (87)	59 (87)	11 (79)	38 (95)	44 (94)	60 (95)	46 (92)	44 (94)	27 (90)	40 (95)	32 (86)	0.51
***Radiology***														
**X-Ray**	511 (100, N.E)	34 (100)	39 (100)	68 (100)	14 (100)	40 (100)	47 (100)	63 (100)	50 (100)	47 (100)	30 (100)	42 (100)	37 (100)	N.E.
**Computed Tomography**	277 (54, 49–59)	28 (82)	21 (54)	24 (35)	6 (43)	28 (70)	19 (40)	25 (40)	21 (42)	34 (72)	2 (7)	40 (95)	29 (78)	<0.01
**Magnetic Resonance Imaging**	302 (63, 58–68)	29 (85)	15 (38)	49 (72)	9 (64)	24 (60)	30 (64)	23 (37)	23 (46)	29 (62)	6 (20)	40 (95)	25 (68)	<0.01
**Ultrasound**	501 (98, 97–99)	34 (100)	36 (92)	68 (100)	14 (100)	40 (100)	47 (100)	62 (98)	47 (94)	46 (98)	30 (100)	41 (98)	36 (97)	0.17
***Endoscopy***														
**Gastroscopy**	376 (72, 67–77)	26 (76)	33 (85)	52 (76)	8 (57)	33 (82)	30 (64)	47 (75)	31 (62)	32 (68)	26 (87)	27 (64)	31 (84)	0.05
**Flexible Sigmoidoscopy**	226 (42, 37–48)	15 (44)	13 (33)	28 (41)	6 (43)	27 (68)	19 (40)	37 (59)	11 (22)	25 (53)	18 (60)	13 (31)	14 (38)	<0.01
**Colonoscopy**	185 (32, 28–37)	9 (26)	16 (41)	21 (31)	3 (21)	22 (55)	7 (15)	32 (51)	13 (26)	27 (57)	13 (43)	10 (24)	12 (32)	<0.01

0–50% shaded red, 51–75% shaded amber, 76–100% shaded green.

N.E.–Not Estimable

Regions: C.Mid—Central Midlands; Manc- Cheshire, Lancashire and Greater Manchester; N.East- Cumbria and North-East; East- East; Lndn-London; N.Mid- North Midlands; Sth.C-South Central; Sth.E- South East; Wessx- Wessex; W.Mid- West Midlands; YksH- Yorkshire and Humber.

### Laboratory tests

GPs reported having the poorest access to testing for occult blood in faeces (54%, 95% CI 49–59) with significant variation between regions (range 34–69%, p<0.01) (Table 1). Access to Urine Protein Electrophoresis was also incomplete (89%, 95% CI 84–92%) but without significant variation between regions. Nearly all GPs reported direct access to the remaining laboratory tests with no significant between-region variation. Full Blood Count, Renal Function, and Liver Function are not reported in Table 1 as all GPs reported having direct access.

### Radiology

Almost all GPs reported having direct access to X-Ray and Ultrasound. The quickest request to test (R-T) time was for X-Ray with most GPs reporting same day testing and getting the result within 1 week. Consequently, two-thirds (68%, 95% CI 63–73%) of GPs reported receiving results within 2 weeks of the request by using a routine referral, and requesting urgent referral was no quicker (Table 2). The greatest variation in access between regions was for CT (7–95%, p<0.01) and MRI (20–95%, p<0.01) (Table 1). The modal routine request-to-test (R-T) and test-to-result (T-R) intervals for most radiological tests was 2–4 weeks and 1–2 weeks respectively, reducing to 1–2 weeks and <1 weeks for urgent referrals. The proportion of GPs receiving radiology results other than X-ray within 2 weeks was low—ranging from 5% (MRI) to 20% (ultrasound).

**Table 2:** Time intervals for tests for GP Direct Access Imaging and Endoscopy in England.

TEST	Request to Test (R-T)							Test to Report (T-R)							Request to Report (R-R) <2 weeks
	n (%)							n (%)							
(N = 511)	Same day	<1 week	1–2 weeks	2–4 weeks	4–6 weeks	>6 weeks	No DA or DK	Same day	<1 week	1–2 weeks	2–4 weeks	4–6 weeks	>6 weeks	No DA or DK	% (95% CI)
***Routine***															
**X-ray**	288 (56)	111 (22)	61 (12)	36 (7)	11 (2)	0 (0)	4 (1)	28 (5)	264 (52)	181 (35)	31 (6)	3 (1)	2 (0)	2 (0)	68 (63–73)
**Computed Tomography**	0 (0)	5 (1)	29 (6)	99 (19)	92 (18)	35 (7)	251 (49)	4 (1)	94 (18)	119 (23)	43 (8)	8 (2)	0 (0)	243 (48)	1 (0–3)
**Magnetic Resonance Imaging**	0 (0)	2 (0)	16 (3)	117 (23)	105 (21)	57 (11)	214 (42)	1 (0)	103 (20)	133 (26)	44 (9)	14 (3)	2 (0)	214 (42)	0 (0–2)
**Ultrasound**	0 (0)	9 (2)	78 (15)	213 (42)	149 (29)	48 (9)	14 (3)	21 (4)	243 (48)	189 (37)	32 (6)	6 (1)	2 (0)	18 (4)	2 (1–4)
**Gastroscopy**	0 (0)	1 (0)	14 (3)	137 (27)	142 (28)	73 (14)	144 (28)	9 (2)	141 (28)	165 (32)	43 (8)	2 (0)	3 (1)	148 (29)	1 (0–2)
**Flexible Sigmoidoscopy**	0 (0)	2 (0)	11 (2)	81 (16)	83 (16)	39 (8)	295 (58)	4 (1)	82 (16)	107 (21)	20 (4)	1 (0)	1 (0)	296 (58)	0 (0–1)
**Colonoscopy**	0 (0)	1 (0)	8 (2)	52 (10)	66 (13)	41 (8)	343 (67)	4 (1)	62 (12)	80 (16)	18 (4)	4 (1)	1 (0)	342 (67)	0 (0–1)
***Urgent***															
**X-ray**	264 (52)	65 (13)	17 (3)	0 (0)	0 (0)	0 (0)	165 (32)	119 (23)	191 (37)	34 (7)	2 (0)	0 (0)	0 (0)	165 (32)	64 (58–69)
**Computed Tomography**	3 (1)	48 (9)	82 (16)	28 (5)	6 (1)	1 (0)	343 (67)	26 (5)	108 (21)	32 (6)	6 (1)	0 (0)	0 (0)	339 (66)	11 (8–14)
**Magnetic Resonance Imaging**	1 (0)	26 (5)	70 (14)	25 (5)	8 (2)	0 (0)	381 (75)	13 (3)	85 (17)	30 (6)	8 (2)	1 (0)	0 (0)	374 (73)	5 (4–8)
**Ultrasound**	7 (0)	105 (5)	138 (14)	39 (5)	8 (2)	1 (0)	213 (75)	59 (12)	189 (37)	48 (9)	0 (0)	1 (0)	0 (0)	214 (42)	20 (17–24)
**Gastroscopy**	0 (0)	11 (5)	87 (14)	53 (5)	3 (2)	2 (0)	355 (75)	18 (4)	91 (18)	46 (9)	6 (1)	0 (0)	0 (0)	350 (68)	5 (3–8)
**Flexible Sigmoidoscopy**	1 (0)	6 (5)	50 (14)	28 (5)	2 (2)	1 (0)	423 (75)	11 (2)	52 (10)	28 (5)	2 (0)	0 (0)	0 (0)	418 (82)	2 (1–4)
**Colonoscopy**	0 (0)	5 (5)	42 (14)	25 (5)	1 (2)	1 (0)	437 (75)	8 (2)	44 (9)	25 (5)	2 (0)	0 (0)	0 (0)	432 (85)	2 (1–3)

DA = Direct Access. DK = Don’t Know. Modal values are shaded blue.

### Endoscopy

Access to endoscopy was poor overall, ranging from 32% (95% CI 28–37%) for colonoscopy to 72% (67–77%)) for gastroscopy, with significant variation between regions for colonoscopy (range 15–57%, p<0.01) and flexible sigmoidoscopy (range 22–68%, p<0.01). The modal time from request to test (R-T) was 4–6 weeks for all routine endoscopy requests, reducing to 1–2 weeks for an urgent request. The modal test-to-result (T-R) intervals were similar to radiology (excluding X-ray) for routine and urgent requests but far fewer GPs reported receiving the result within 2 weeks of the request, ranging from 2% (colonoscopy) to 5% (gastroscopy).

### Direct Access in relation to the 2015 NICE guideline recommendations

Table 3 shows that at the time of the survey the vast majority of GPs could not directly access the tests recommended in the 2015 NICE guidance. In some cases (ultrasound, MRI, gastroscopy) the proportion who could was very low (6% or less). There was very little variation reported between regions, except for direct access to CT within 2 weeks (range 0–25%, p<0.01).

**Table 3:** Number (%) of English GPs who can directly access tests as recommended in the 2015 NICE guidance by region.

Test	ENGLAND N = 511	REGION n (%)												
	n (%, 95 C.I)*	C.Mid (N = 34)	East (N = 39)	Lndn (N = 68)	Manc (N = 14)	N.East (N = 40)	N.Mid (N = 47)	Sth.C (N = 63)	Sth.E (N = 50)	Sth.W (N = 47)	Wessx(N = 30)	W.Mid (N = 42)	YksH (N = 37)	*p*-value
***NICE 2015 recommends GP direct access <48 hours***														
**X-ray**	99 (19, 16–24)	6 (18)	8 (21)	18 (26)	2 (14)	15 (38)	11 (23)	6 (10)	11 (22)	9 (19)	2 (7)	4 (10)	7 (19)	0.04
**Ultrasound**	6 (1, 0–2)	0 (0)	0 (0)	0 (0)	0 (0)	1 (2)	0 (0)	1 (2)	2 (4)	1 (2)	0 (0)	0 (0)	1 (3)	0.66
***NICE 2015 recommends GP direct access <2 weeks***														
**X-ray**	442 (88, 86–91)	31 (91)	31 (79)	62 (91)	14 (100)	36 (90)	45 (96)	52 (83)	47 (94)	38 (81)	23 (77)	31 (74)	32 (86)	0.02
**Computed Tomography**	58 (11, 8–15)	8 (24)	6 (15)	4 (6)	1 (7)	10 (25)	6 (13)	3 (5)	3 (6)	6 (13)	0 (0)	3 (7)	8 (22)	<0.01
**Magnetic Resonance Imaging**	29 (6, 4–8)	5 (15)	4 (10)	4 (6)	0 (0)	3 (8)	3 (6)	1 (2)	3 (6)	1 (2)	0 (0)	2 (5)	3 (8)	0.11
**Ultrasound**	126 (22, 18–25)	7 (21)	11 (28)	16 (24)	0 (0)	14 (35)	15 (32)	8 (13)	22 (44)	10 (21)	9 (30)	7 (17)	7 (19)	0.01
**Gastroscopy**	23 (5, 3–8)	2 (6)	3 (8)	2 (3)	1 (7)	5 (12)	0 (0)	3 (5)	0 (0)	1 (2)	3 (10)	1 (2)	2 (5)	0.11

0–50% shaded red, 51–75% shaded amber, 76–100% shaded green.

Regions: C.Mid—Central Midlands; Manc- Cheshire, Lancashire and Greater Manchester; N.East- Cumbria and North-East; East- East; Lndn-London; N.Mid- North Midlands; Sth.C-South Central; Sth.E- South East; Wessx- Wessex; W.Mid- West Midlands; YksH- Yorkshire and Humber.

*National estimates represent weighted averages, weighted by the inverse of the sampling fraction (number sampled within region/Number of practices in region)

### Results reporting

The reported test-to-result (T-R) interval suggests that only X-rays results are received within the time interval recommended by NICE. To overcome this, many GPs reported that if an abnormality that could be cancer was found on investigation 90.7% would receive a fax of the result; 41.8% reported that that the result would be communicated by the standard computerised system results linkage, 26.4% would receive a telephone call, and 3.8% would receive and email.

## Discussion

### Main findings

Our findings show that English GPs could not access directly many of the diagnostic tests recommended by the 2015 NICE guidance for suspected cancer when it was released, with significant regional variation in direct test access. The vast majority of English GPs were unable to directly access imaging and endoscopy (and receive a report) within the intervals recommended by NICE, regardless of the urgency of the test request. Whilst almost all GPs reported direct access to X-ray and ultrasound, the proportion with access within 2 weeks dropped to 88% and 22% respectively (19% and 1% respectively for access within 48 hours). For the remaining imaging and endoscopy tests, direct access ranged from 32% (colonoscopy) to 72% (gastroscopy) dropping to between 5% (Gastroscopy) and 11% (CT) if restricted to access within the time intervals specified by NICE.

Significant inter-regional variation was reported for the majority of imaging and endoscopic tests; those without significant regional variation had the lowest levels of timely access (0–15%). Some GPs reported that the length of the request to test (R-T) interval was not associated with the test to report (T-R) interval: a rapid test could be twinned with a longer reporting interval resulting in a request to report (R-R) interval longer than 2 weeks. To mitigate this, most GPs reported there was a system in place (a fax in 91%) to expedite the communication of positive findings, but this approach is flawed: firstly the individual conducting the test must recognise that serious pathology might be present to expedite the report, and secondly for the tests that GPs reported greatest direct access (CXR and ultrasound) a negative result does not definitively exclude cancer [14, 15]. Whatever the outcome of the test, GPs require rapid reporting to prevent any delay in further investigation or referral for ongoing symptoms or concern [5].

In contrast, access to laboratory testing was uniformly good but with two notable exceptions: testing for occult blood in faeces and urine electrophoresis. The use of faecal occult blood testing (FOBT) has been opposed by hospital specialists as the older qualitative guaiac-based method has poor sensitivity when used in symptomatic primary care patients, and so a high rate of false negatives and false reassurance. This provides some explanation for why FOBT had gone out of favour in many hospital laboratories across NHS England prior to NICE 2015 [16]. However, following superior diagnostic performance in the screening setting, evidence is accumulating for the superior diagnostic accuracy of quantitative Faecal Immunochemical Testing (FIT) for primary care patients with lower abdominal symptoms: in one study a negative predictive value of 100% for colorectal cancer [17] and some laboratories are already moving to adopt FIT, especially given the added relative simplicity of sampling and absence of dietary restrictions [18]. Urine protein electrophoresis has also been superseded in some laboratories (and in the International Myeloma Working Group (IMWG) and draft 2015 NICE Myeloma guidelines) by the Freelite assay [19, 20] which can detect serum free light-chains (Bence-Jones proteins) in blood, avoiding the need for urine samples which are only returned by around 40% of patients [21].

### Limitations

The key limitation of the survey is that we used a purposive sampling strategy rather than random sampling. The GPs invited to participate were selected by local CRN leads (so that the actual response rate is difficult to characterise) and 44% of GPs failed to complete the survey after opening it (and we have no indication why). The justification for our purposive sampling strategy was that we were likely to get a very low response rate to a random sample and would have no guarantee of data quality. We felt we would get nearer the truth by recruiting local research practices committed to providing accurate and precise information. We also recognised that test access in the NHS is dependent on the location of the GP practice so obtaining a representative sample of individual GPs was less important. However, our method is likely to give a more reliable estimate than random sampling only if the research network GPs that did respond gave a well informed and unbiased report of local access. This may not have been the case. We did not pre-specify whether GPs should respond to the survey using written evidence (e.g. clinical records or local guidelines) or from memory. GPs who responded from memory may have been influenced by memorable positive or negative experiences of test access. Consequently, the generalisability of our findings must be interpreted with caution.

The fact that the results are consistent with the national Diagnostic Imaging Dataset (see below) provides some reassurance. However, we also observed occasional inconsistencies within the survey data—for example, a small number of respondents said they had no direct access to Ca-125 or serum protein electrophoresis. This is unlikely to be true as both are routine tests in hospital laboratories. Ca-125 has been recommended for use in primary care by NICE since 2011 and serum protein electrophoresis is routinely done in response to requests for immunoglobulin or myeloma screens. Similarly, a few respondents reported that they had no direct access to specific tests but subsequently completed the section on how long it took to request the test and receive the results. However, to maximize internal validity, we excluded them from the analysis.

### Previous literature

Until recently there have been very few data on GP direct access to diagnostic services in English primary care. The most recent is the Diagnostic Imaging Dataset (DID) which reports the “imaging activity in patients directly referred by a GP” for NHS England in the same time period as our survey (April-Jun 2015) [22]. DID is a central collection of detailed information about diagnostic imaging tests carried out on NHS patients, extracted from local Radiology Information Systems (RISs) and submitted monthly, but does not discriminate between whether a test request is made for suspected cancer or another clinical purpose, or collect data on Endoscopy, or specify the urgency of the request [23]. However, the median time for both the request to test (R-T) and test to report (T-R) interval was consistent with our findings. For example, for brain MRI the median R-T interval reported by DID was 21–25 days (our median category was 4–6 weeks for routine and 1–2 weeks urgent MRI) and the T-R interval was reported as 2 days (our median interval was 1–2 weeks routine and <1 week for urgent MRI). Similarly for CT of the chest or abdomen, DID reported median R-T and T-R intervals of 15–19 days and 3–3.5 days respectively. Our median R-T and T-R intervals were 2–4 weeks and 1–2 week respectively for routine CT and 1-2weeks and <1 week for urgent CT.

Our data are also consistent with the ICBP3 survey data of 251 English Primary Care Practitioners between May 2012 and July 2013: 94% reported direct access to laboratory tests (but did not specify which); 68.1% (61.9–73.8) to gastroscopy; 40.6% (34.6–47.0) to flexible sigmoidoscopy; and 33.1 (27.4–39.3) to colonoscopy [1]. However, the ICBP3 practitioners reported significantly poorer overall access to radiology and ultrasound: X-Ray 82.5% (77.1–86.9); CT 21.5% (16.7–27.2); MRI 19.9% (15.3–25.5) and ultrasound 78.1% (72.4–82.9. Following the ICBP3 survey, Macmillan conducted a survey in 2014 of the GPs they employ to facilitate cancer care[24]. Direct access was reported as 100% for CxR (73% same day), 50% for brain MRI (none same day), and 41% for flexible sigmoidoscopy (none same week). As with our survey, there was considerable variation in access with 12% reporting they could get a pelvic ultrasound in < 1week but 18% reporting a wait of 2–4 weeks, with 58% saying they waited >1 week after the scan for a report. In the same year a survey of CCGs reported that 25%, 27%, 49%, and 50% of CCGs were not offering direct access CxR, Pelvic Ultrasound, MRI Brain, and Flexible Sigmoidoscopy, respectively [25].

These trends imply improvements in direct access to radiology (but not endoscopy) in the two years following July 2013. Recent reports confirm that imaging activity is growing by 6% per annum, with CT and MRI projected to increase by 9% per annum in the future, yet equipment and workface shortfalls currently restrict capacity [26]. Variations in request to test and test to report intervals warrant further investigation to understand how workforce and equipment shortfalls could be mitigated in the presence of suggested drivers of increasing demand: demographic change, new clinical guidelines, lowered scanning thresholds and widening indications, increased surveillance in chronic disease and cancer survivors, and increased use of screening. Potential solutions, such as radiology reporting networks, should be evaluated and best practice shared [26]. At the time of writing, endoscopy was predicted to increase to 750,000 procedures by 2020 (a 44% increase), but gaps in workforce, efficiency, and data quality are likely to (continue to) prevent uniform direct and rapid access [27].

### Implications

Our data will be of use to policymakers and CCGs during the ongoing remodelling of NHS services in response to the 2015 NICE guidance as they will provide a baseline for follow-up studies investigating changes in test access following the guideline’s introduction, and hopefully act as a catalyst for investment in improved GP direct access. They will inform the ongoing evaluation of novel pathways to diagnosis such as the Accelerate Coordinate and Evaluate (ACE) programme which is underway in England to understand whether the Danish Multidisciplinary Diagnostic Centre (MDC) model can be replicated in England as an alternative approach to swift comprehensive assessment of lower-risk but not no-risk symptoms such as weight loss and fatigue [28–32]. For this type of pathway to succeed there needs to be rapid up-front access to diagnostic tests (in Denmark patients are offered USS/CT and laboratory tests within 1 week): this is clearly not possible without significantly changing the structure of current NHS diagnostic pathways [29].

As programmes such as ACE are rolled out, robust evaluation needs to identify the resources required to increase test access, to understand the displacement of patients from other urgent and routine referral pathways, and to ascertain the most appropriate test providers, whilst documenting the salient diagnostic pathway intervals as set out in the Aarhus statement [33]. As NHS regions respond to the 2015 NICE guidelines repeat evaluations of GPs access to tests should be conducted with similar surveys performed across the NHS and comparable developed nations, in particular those where GPs also operate a gatekeeper role to investigations and specialist review [34, 35].

## Supporting Information

S1 Appendix NICE 2015 recommendations for primary care testing. (DOCX)

S2 Appendix GP reported Request to Test (R-T) interval in relation to the Test to Report (T-R) interval by routine test. (DOCX)

S1 Survey Instrument Copy of the GP survey. (PDF)
