# Supplementary material for: Variation in Direct Access to Tests to Investigate Cancer: A Survey of English General Practitioners
Source: PLoS One. 2016 Jul 22;11(7):e0159725. doi: 10.1371/journal.pone.0159725 (PMC4957804; doi:10.1371/journal.pone.0159725)
Supplement: S1 Appendix — (DOCX) [file pone.0159725.s001.docx]

**S1 Appendix**- NICE 2015 recommendations for primary care testing.

| **NICE 2015 Recommendations for Direct Access** | **Occurrences (n)** |
| --- | --- |
| Blood Tests (unspecified) | 2 |
| Ca-125 | 9 |
| Computed Tomography or Magnetic Resonance Imaging within 2 weeks | 1 |
| Computed Tomography within 2 weeks | 8 |
| Faecal Occult Blood Testing | 5 |
| Full Blood Count within 48 hours | 2 |
| Gastroscopy (Non-Urgent) | 9 |
| Gastroscopy within 2 weeks | 5 |
| Ultrasound (Not specified) | 7 |
| Ultrasound within 2 weeks | 5 |
| Ultrasound within 48 hours | 1 |
| X-Ray within 2 weeks | 28 |
| X-Ray within 48 hours | 2 |
