# Supplementary material for: Variation in Direct Access to Tests to Investigate Cancer: A Survey of English General Practitioners
Source: PLoS One. 2016 Jul 22;11(7):e0159725. doi: 10.1371/journal.pone.0159725 (PMC4957804; doi:10.1371/journal.pone.0159725)
Supplement: S2 Appendix — (DOCX) [file pone.0159725.s002.docx]

**S2 Appendix–** GP reported Request to Test (R-T) interval in relation to the Test to Report (T-R) interval by routine test.

|  | **Test to result (T-R)** | | | | | | | | | | | | |
| --- | --- | --- | --- | --- | --- | --- | --- | --- | --- | --- | --- | --- | --- |
|  | **X-ray** | *< 1w* | *1-2w* | *2-4w* | *4-6w* | *> 6w* |  | **USS** | *< 1w* | *1-2w* | *2-4w* | *4-6w* | *> 6w* |
| **Request to test (R-T)** | *< 1w* | 236 | 136 | 23 | 3 | 0 |  | *< 1w* | 7 | 2 | 0 | 0 | 0 |
|  | *1-2w* | 33 | 25 | 3 | 0 | 0 |  | *1-2w* | 53 | 20 | 3 | 0 | 0 |
|  | *2-4w* | 15 | 17 | 3 | 0 | 0 |  | *2-4w* | 119 | 79 | 11 | 1 | 0 |
|  | *4-6w* | 5 | 3 | 1 | 0 | 2 |  | *4-6w* | 70 | 62 | 11 | 3 | 0 |
|  | *> 6w* | 0 | 0 | 0 | 0 | 0 |  | *> 6w* | 13 | 24 | 7 | 2 | 2 |
|  | **Gastroscopy** | | | | | |  | **CT** | | | | | |
|  | *< 1w* | 1 | 0 | 0 | 0 | 0 |  | *< 1w* | 3 | 1 | 1 | 0 | 0 |
|  | *1-2w* | 9 | 3 | 1 | 0 | 0 |  | *1-2w* | 16 | 10 | 3 | 0 | 0 |
|  | *2-4w* | 71 | 56 | 6 | 1 | 0 |  | *2-4w* | 37 | 46 | 13 | 2 | 0 |
|  | *4-6w* | 45 | 75 | 17 | 0 | 0 |  | *4-6w* | 27 | 43 | 18 | 4 | 0 |
|  | *> 6w* | 22 | 29 | 17 | 1 | 3 |  | *> 6w* | 12 | 16 | 5 | 1 | 0 |
|  | **Colonoscopy** | | | | | |  | **MRI** | | | | | |
|  | *< 1w* | *1* | 0 | 0 | 0 | 0 |  | *< 1w* | 1 | 0 | 1 | 0 | 0 |
|  | *1-2w* | *6* | 1 | 1 | 0 | 0 |  | *1-2w* | 14 | 2 | 0 | 0 | 0 |
|  | *2-4w* | *25* | 22 | 2 | 1 | 0 |  | *2-4w* | 42 | 55 | 13 | 6 | 0 |
|  | *4-6w* | *24* | 32 | 8 | 0 | 0 |  | *4-6w* | 26 | 50 | 23 | 3 | 1 |
|  | *> 6w* | *8* | 22 | 7 | 2 | 1 |  | *> 6w* | 19 | 26 | 6 | 5 | 1 |
|  | **Flexible Sigmoidoscopy** | | | | | |  | Shaded region indicates whether request to test time is the same as or shorter than test to result time. | | | | | |
|  | *< 1w* | *2* | 0 | 0 | 0 | 0 |  |  |  |  |  |  |  |
|  | *1-2w* | *6* | 4 | 1 | 0 | 0 |  |  |  |  |  |  |  |
|  | *2-4w* | *40* | 35 | 3 | 1 | 0 |  |  |  |  |  |  |  |
|  | *4-6w* | *30* | 45 | 7 | 0 | 0 |  |  |  |  |  |  |  |
|  | *> 6w* | *7* | 23 | 8 | 0 | 1 |  |  |  |  |  |  |  |
