# Supplementary material for: Variation in Direct Access to Tests to Investigate Cancer: A Survey of English General Practitioners
Source: PLoS One. 2016 Jul 22;11(7):e0159725. doi: 10.1371/journal.pone.0159725 (PMC4957804; doi:10.1371/journal.pone.0159725)
Supplement: S1 Survey Instrument — (PDF) [file pone.0159725.s003.pdf]

GP access to diagnostic tests for cancer

Survey information and consent

Many thanks for agreeing to take part in this survey of GP access to diagnostic tests for cancer in NHS England.

"Direct access" is defined as a test that a GP can request without having to speak to a specialist.

The survey includes questions about access to the following tests: blood tests; endoscopy; Xray; Computed Tomography (CT/PET); Magnetic Resonance Imaging (MRI); Ultrasound (USS).

An example of a test provider is a hospital trust, whereas an example of a test centre is a hospital.

This is a survey funded by Cancer Research UK, designed by Oxford University and administered by the NHS National Institute of Health Research Clinical Research Network (NIHR CRN).

The survey has been reviewed by, and received ethics clearance through, the University of Oxford Central University Research Ethics Committee. All data collected will be stored anonymously and securely on the Oxford University server, and will be made available to Cancer Research UK and other researchers following an application and approval process.

The point of contact for any questions, queries or complaints is Brian.Nicholson@phc.ox.ac.uk

*1. Please tick the following box to confirm that you consent to take part in the survey

- I consent
- I consent (but opt-out of sharing identifiable practice level data)
- I do not consent

Practice information

***2. In which CCG is your GP practice situated?**

***3. Please tell us the name, postcode, and size of your GP practice?**

(These data will not be shared if you have opted out)

Practice name

Postcode

Size (patient population)

***4. How many NHS test providers do you have access to?**

(An example of a test provider is a hospital trust)

Please name the NHS test providers

***5. How many NHS test centres do you have access to?**

(An example of a test centre is a hospital within a hospital trust)

Please name the NHS test centres

***6. How many private test providers do you access?**

Please specify the provider and corresponding tests

***7. In your understanding, are arrangements for test access the same across all GP practices in your CCG?**

- Yes
- No
- Don't know

Comments

Test Access

"Direct access" is defined as a test that a GP can request without having to speak to a specialist.

***8. Please tell us if you agree with the following statement: "In our practice, we have direct access to the following laboratory tests commonly used during cancer investigation"**

	Yes	No	Don't know
Ca125	○	○	○
Calcium	○	○	○
Erythrocyte Sedimentation Rate	○	○	○
Faecal Occult Blood Test	○	○	○
Full Blood Count	○	○	○
Liver Function Tests	○	○	○
Plasma Viscosity	○	○	○
PSA	○	○	○
Renal Function	○	○	○
Serum Protein Electrophoresis	○	○	○
Urine Protein Electrophoresis	○	○	○

Comments:

GP access to diagnostic tests for cancer

***9. What options do you have to access the following investigations? (Please check all that apply)**

("Direct access" is defined as a test that a GP can request without having to speak to a specialist.)

	GP direct access- routine	GP direct access- urgent	2 Week Wait referral	One-stop clinic referral (where the test would be performed)	Refer to specialist (who would then request the test)	Don't know
X Ray	☐	☐	☐	☐	☐	☐
CT Scan	☐	☐	☐	☐	☐	☐
MRI Scan	☐	☐	☐	☐	☐	☐
Ultrasound scan	☐	☐	☐	☐	☐	☐
PET Scan	☐	☐	☐	☐	☐	☐
Upper GI Endoscopy	☐	☐	☐	☐	☐	☐
Flexible Sigmoidoscopy	☐	☐	☐	☐	☐	☐
Colonoscopy	☐	☐	☐	☐	☐	☐
Mammography	☐	☐	☐	☐	☐	☐

Comments:

***10. I have direct access to the following investigations, without having to refer to a specialist first:**

(Please check all that apply, and select N/A if you are not able to order these tests directly)

	Brain	Thorax	Abdomen	Pelvis	Soft tissue / musculoskeletal	N/A
XRay	☐	☐	☐	☐	☐	☐
CT	☐	☐	☐	☐	☐	☐
MRI	☐	☐	☐	☐	☐	☐
USS	☐	☐	☐	☐	☐	☐
PET	☐	☐	☐	☐	☐	☐

Please comment:

***11. For the investigations you have direct access, what is the average waiting time for a ROUTINE test to be performed?**

(not including 2WW, one-stop, or specialist referrals)

(Please select N/A if you are not able to order these tests directly)

	Same day	<1 week	1-2 weeks	2-4 weeks	4-6 weeks	6-12 weeks	>12 weeks	N/A
X Ray	○	○	○	○	○	○	○	○
CT	○	○	○	○	○	○	○	○
MRI scan	○	○	○	○	○	○	○	○
Ultrasound scan	○	○	○	○	○	○	○	○
Upper GI endoscopy	○	○	○	○	○	○	○	○
Flexible sigmoidoscopy	○	○	○	○	○	○	○	○
Colonoscopy	○	○	○	○	○	○	○	○
Mammography	○	○	○	○	○	○	○	○
PET	○	○	○	○	○	○	○	○

Please explain if the waiting time varies between body sites:

***12. From the time the investigation is performed, how quickly are ROUTINE results usually communicated to you?**

(Please select N/A if you are not able to order these tests directly)

	Same day	<1 week	1-2 weeks	2-4 weeks	4-6 weeks	6-12 weeks	>12 weeks	N/A
X Ray	○	○	○	○	○	○	○	○
CT	○	○	○	○	○	○	○	○
MRI scan	○	○	○	○	○	○	○	○
Ultrasound scan	○	○	○	○	○	○	○	○
Upper GI endoscopy	○	○	○	○	○	○	○	○
Flexible sigmoidoscopy	○	○	○	○	○	○	○	○
Colonoscopy	○	○	○	○	○	○	○	○
Mammography	○	○	○	○	○	○	○	○
PET	○	○	○	○	○	○	○	○

Please explain if these times vary between body sites:

GP access to diagnostic tests for cancer

***13. For the investigations you have direct access, what is the average waiting time for an URGENT test to be performed?**

(not including 2WW, one-stop, or specialist referrals)

(Please select N/A if you are not able to order these tests directly)

	Same day	<1 week	1-2 weeks	2-4 weeks	4-6 weeks	6-12 weeks	>12 weeks	N/A
X Ray	○	○	○	○	○	○	○	○
CT	○	○	○	○	○	○	○	○
MRI scan	○	○	○	○	○	○	○	○
Ultrasound scan	○	○	○	○	○	○	○	○
Upper GI endoscopy	○	○	○	○	○	○	○	○
Flexible sigmoidoscopy	○	○	○	○	○	○	○	○
Colonoscopy	○	○	○	○	○	○	○	○
Mammography	○	○	○	○	○	○	○	○
PET	○	○	○	○	○	○	○	○

Please explain if the waiting time varies between body sites:

***14. From the time the investigation is performed, how quickly are URGENT results usually communicated to you?**

(Please select N/A if you are not able to order these tests directly)

	Same day	<1 week	1-2 weeks	2-4 weeks	4-6 weeks	6-12 weeks	>12 weeks	N/A
X Ray	○	○	○	○	○	○	○	○
CT	○	○	○	○	○	○	○	○
MRI scan	○	○	○	○	○	○	○	○
Ultrasound scan	○	○	○	○	○	○	○	○
Upper GI endoscopy	○	○	○	○	○	○	○	○
Flexible sigmoidoscopy	○	○	○	○	○	○	○	○
Colonoscopy	○	○	○	○	○	○	○	○
Mammography	○	○	○	○	○	○	○	○
PET	○	○	○	○	○	○	○	○

Please explain if these times vary between body sites:

***15. When an investigation finds an abnormality that could be cancer, how is this result communicated back to you?**

☐ Usual results linkage

☐ Telephone call

☐ Fax of report

☐ Email

☐ Letter

☐ Other (please specify)

***16. What local or national guidance in relation to test choice do you follow?**

○ There are no guidelines

○ There are guidelines but I don't follow them

○ I follow guidelines (please specify)

***17. In the past 3 years, has there been any change to test access in your CCG?**

○ Yes

○ No

○ Don't know

Comments:

Specialist access

***18. Please rank agreement with the following statement (does not include advice from on-call registrars).**

"I can obtain consultant level advice in less than 48 hours regarding investigations for suspected cancer"

1 (strongly disagree)	2	3	4	5 (strongly agree)
○	○	○	○	○

***19. Please rank agreement with the following statement (does not include advice from on-call registrars).**

"I can obtain consultant level advice in less than 48 hours regarding potential referrals to secondary care/specialist care services for suspected cancer"

1 (strongly disagree)	2	3	4	5 (strongly agree)
○	○	○	○	○

***20. In your judgement, do more than half of your patients live more than 25 miles from their local hospital with specialist cancer services?**

- ○ Yes
- ○ No
- ○ Don't know

Final comments

*21. Do you have any further comments about direct access tests?
